# Total pleural coverage followed by lung transplantation in patient with lymphangioleiomyomatosis

**DOI:** 10.1007/s11748-019-01217-0

**Published:** 2019-10-14

**Authors:** Do Hyung Kim, Hyo Yeong Ahn, Bong Soo Son, Joohyung Son

**Affiliations:** 1 Department of Thoracic and Cardiovascular Surgery, Pusan National University Yangsan Hospital, Gyeongnam, South Korea; 2 Department of Thoracic and Cardiovascular Surgery, Medical Research Institution, Pusan National University Hospital, 305, Gudeok-Ro, Seo-Gu, Busan, 602-739 South Korea

**Keywords:** Pneumothorax, Lymphangioleiomyomatosis, Lung transplantation

## Abstract

Tuberous sclerosis complex lymphangioleiomyomatosis (TSC-LAM) is a rare disease, which may develop an intractable pneumothorax. Chemical or mechanical pleurodesis is a general management to prevent recurrence of pneumothorax, rendering it difficult to later dissect the pleura and control intraoperative bleeding. Since total pleural coverage (TPC) alternative to pleurodesis has been firstly reported by Kurihara et al. (Jpn J Thorac Cardiovasc Surg 54:274, 2006), TPC was performed in case of a 46-year-old female with a secondary spontaneous pneumothorax caused by TSC-LAM and followed by lung transplantation. Final pathological report showed the reinforced visceral pleura in the absence of dense adhesions.

## Introduction

Tuberous sclerosis complex lymphangioleiomyomatosis (TSC-LAM) is a rare disease, which may trigger an intractable pneumothorax. Chemical or mechanical pleurodesis is recommended to prevent recurrence, rendering it difficult to later dissect the pleura and control intraoperative bleeding. We performed total pleural coverage (TPC) as an alternative to pleurodesis, based on the recommendations of recent reports. Pathological findings are important when evaluating the efficacy of TPC. Herein, we report the case of a 46-year-old female with a secondary spontaneous pneumothorax caused by TSC-LAM who underwent TPC prior to lung transplantation.

## Case report

**Fig. 1:**
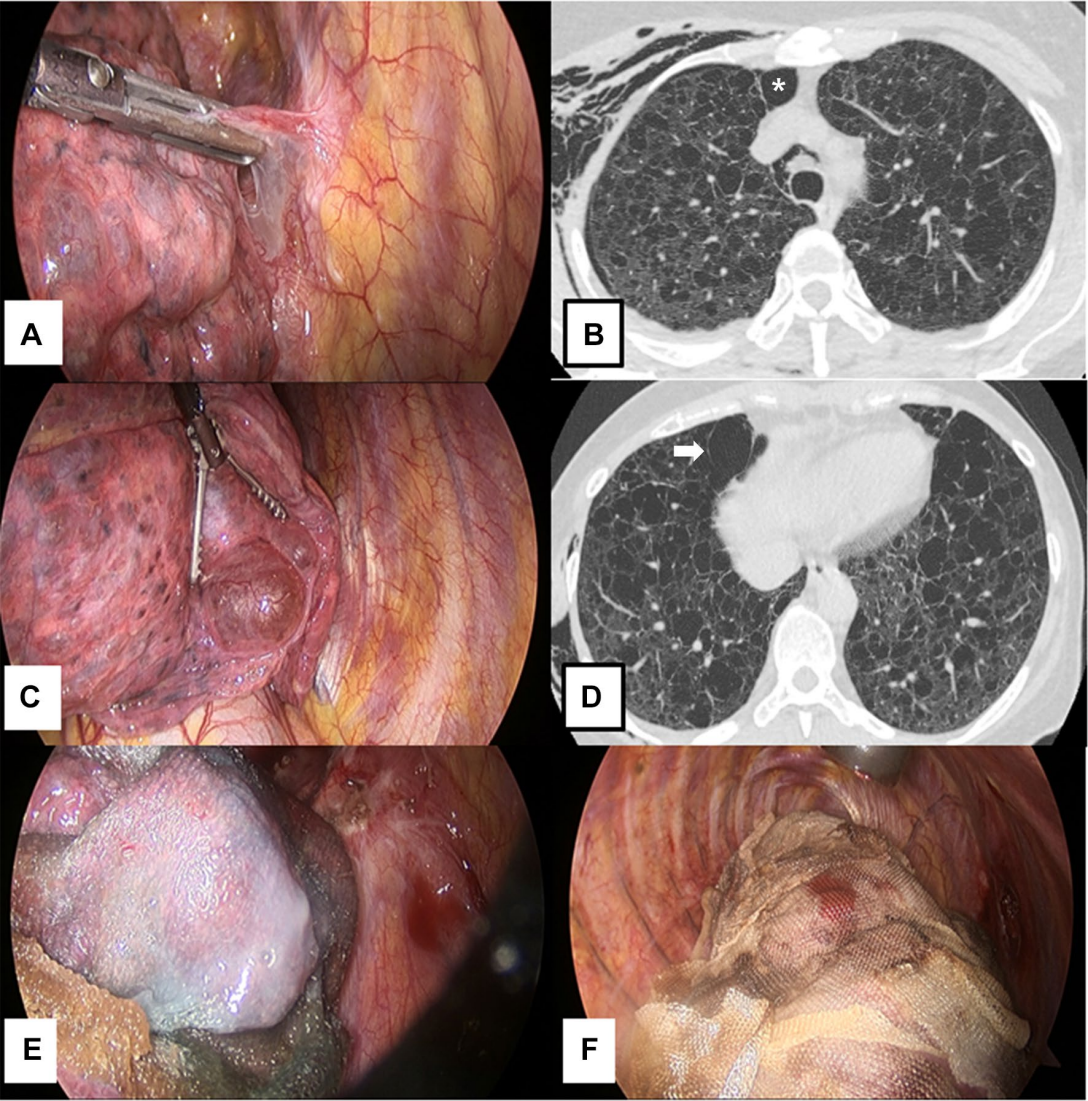
Gross findings of lungs correlated to computed tomographic images. **a** Ruptured bullae were evident in the right upper lobe, as was a dense adhesion around the bullae. The adhesion was released and the bullae loop ligated. **b** The ruptured area (asterisk) contained bullae, as revealed by CT. **c** A huge fragile cyst at risk of immediate rupture was covered by an absorbable polyglycolic acid sheet (**e**). **d** The fragile cyst (arrow) was located near the bullae, as revealed by CT. **f** We performed total pleural coverage (TPC) of the entire lung

A 46-year-old female was admitted to the emergency room with dyspnea, and right pneumothorax was diagnosed. Chest computed tomography (CT) revealed the features of lymphangioleiomyomatosis (LAM), including facial angiofibroma, hypomelanotic macules, and renal angiomyolipoma; we thus diagnosed tuberous sclerosis complex (TSC)-LAM. As the air leakage had developed 10 days prior, airleak control was performed by video-assisted thoracoscopic surgery (VATS), which showed multiple lung cysts with ruptured bullae surrounded by a dense adhesion in the right upper lobe. After releasing the adhesion, the bullae was ligated, and covered a huge fragile cyst at risk of imminent rupture with an absorbable polyglycolic acid sheet (Neoveil; Gunze Ltd., Kyoto, Japan) and fibrin sealant (Tisseel; Baxter Healthcare Corp., Deerfield, IL, USA). To prevent recurrence, we performed total pleural coverage (TPC) of the entire lung surface using 12 sheets of oxidized regenerated cellulose (ORC) mesh (Ethicon SURGICEL ^®^ absorbable Hemostat gauze, Johnson & Johnson, Brunswick, NJ, USA) (Fig. 1). The patient was discharged on postoperative day (POD) 9.

One month later, she was readmitted to treat a contralateral recurrent pneumothorax. She again underwent TPC after ligation of the ruptured bullae and was discharged on POD 22. Home oxygen therapy and sirolimus 1 mg daily was prescribed, because the dyspnea remained aggravated even after operation.

**Fig. 2:**
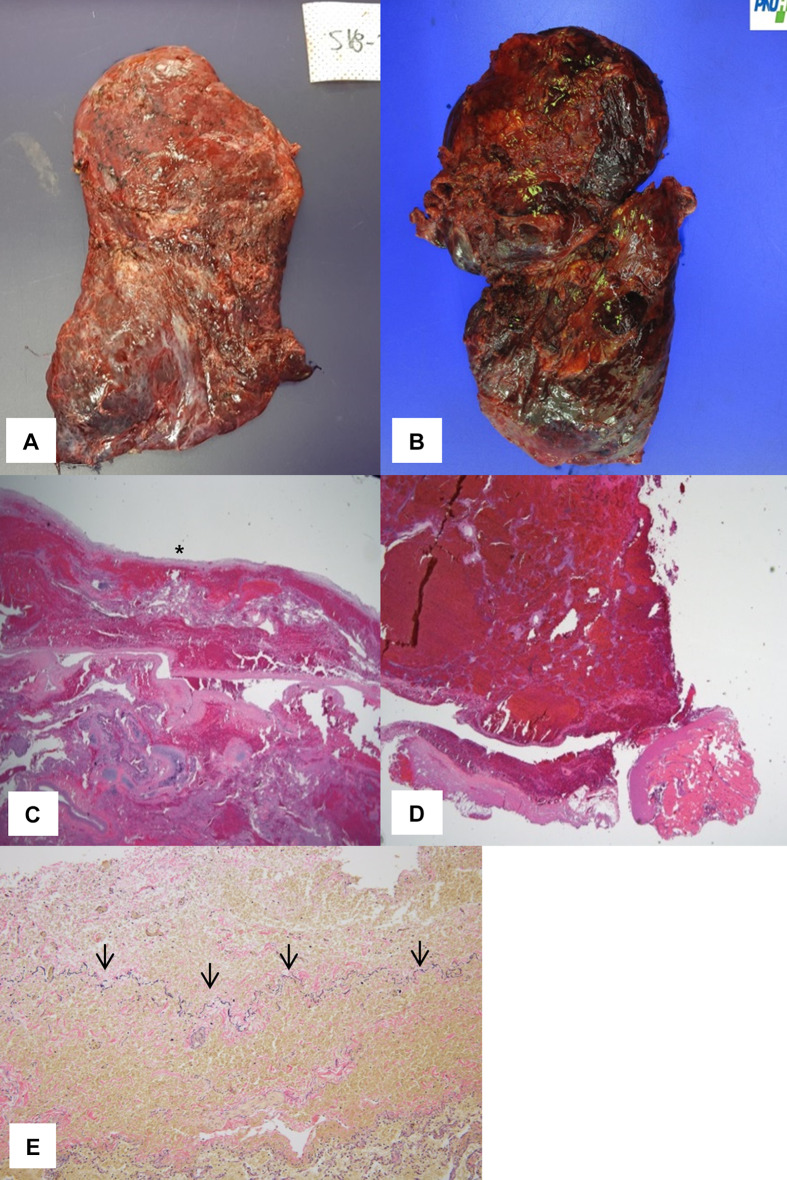
Gross and microscopic findings of lungs that had undergone TPC. **a**, **b** The surfaces of both the right (**a**) and left (**b**) lungs were relatively smooth. **c**, **d** The visceral pleura that had undergone TPC were thickened, and minimal adhesion of the visceral to the parietal pleura was evident. **e** Elastica-Masson stain showed the thickened visceral pleura above the natural visceral pleura (arrow)

Fourteen months later, lung transplantation was successfully performed; both lungs that had been subjected to TPC were sampled, revealing thickened visceral pleura surrounded by minimal inflammation (Fig. 2). After the operation, oxygen therapy was no longer necessary and she was discharged on POD 41 without any complication.

## Discussion

Tuberous sclerosis complex (TSC) is an autosomal-dominant genetic disorder affecting multiple organs. LAM is one manifestation of TSC, caused by a TSC2 mutation triggering aberrant cell proliferation [2].

Since recurrent pneumothorax is one of the most common complications seen in LAM patients, chemical or mechanical pleurodesis has been recommended to prevent recurrence [3, 4]. However, after pleurodesis, it was difficult to dissect the pleura and control intraoperative bleeding, because of severe adhesion. Therefore, TPC was recommended as an alternative to pleurodesis, in which ORC was used to cover the entire pleura to make thickened visceral pleura, as in studies of Noda et al., Kurihara et al., Kusu et al. [3, 5–7].

Although sirolimus inhibits tissue proliferation and the release of lymphangiogenic growth factors [8, 9], and relieves symptoms, the dyspnea worsened in our case as disease progressed, and lung transplantation was performed at 15 months after the initial operation. The pathology showed the thickened pleura without severe inflammation (Fig. 2).

Although animal experiments have been performed to check adhesion or visceral pleural thickness after pleurodesis [3], no report on a patient treated via TPC who later underwent lung transplantation has appeared. As shown here, TPC might be one of the good tools for prevention of recurred pneumothorax, making the thickened visceral pleura useful to describe our patient with LAM who underwent TPC followed by lung transplantation; examination of the extracted lung showed that our treatment reinforced the visceral pleura in the absence of dense adhesions.

## Conclusion

TSC-LAM is a rare disease, which may trigger an intractable pneumothorax. TPC might be one of the good tools for prevention of recurred pneumothorax, making the thickened visceral pleura useful to describe our patient with LAM who underwent TPC followed by lung transplantation.
